# Cryptic Population Dynamics: Rapid Evolution Masks Trophic Interactions

**DOI:** 10.1371/journal.pbio.0050235

**Published:** 2007-09-04

**Authors:** Takehito Yoshida, Stephen P Ellner, Laura E Jones, Brendan J. M Bohannan, Richard E Lenski, Nelson G Hairston

**Affiliations:** 1 Department of Ecology and Evolutionary Biology, Cornell University, Ithaca, New York, United States of America; 2 Department of Biology, University of Oregon, Eugene, Oregon, United States of America; 3 Department of Microbiology and Molecular Genetics, Michigan State University, East Lansing, Michigan, United States of America; Penn State University, United States of America

## Abstract

Trophic relationships, such as those between predator and prey or between pathogen and host, are key interactions linking species in ecological food webs. The structure of these links and their strengths have major consequences for the dynamics and stability of food webs. The existence and strength of particular trophic links has often been assessed using observational data on changes in species abundance through time. Here we show that very strong links can be completely missed by these kinds of analyses when changes in population abundance are accompanied by contemporaneous rapid evolution in the prey or host species. Experimental observations, in rotifer-alga and phage-bacteria chemostats, show that the predator or pathogen can exhibit large-amplitude cycles while the abundance of the prey or host remains essentially constant. We know that the species are tightly linked in these experimental microcosms, but without this knowledge, we would infer from observed patterns in abundance that the species are weakly or not at all linked. Mathematical modeling shows that this kind of cryptic dynamics occurs when there is rapid prey or host evolution for traits conferring defense against attack, and the cost of defense (in terms of tradeoffs with other fitness components) is low. Several predictions of the theory that we developed to explain the rotifer-alga experiments are confirmed in the phage-bacteria experiments, where bacterial evolution could be tracked. Modeling suggests that rapid evolution may also confound experimental approaches to measuring interaction strength, but it identifies certain experimental designs as being more robust against potential confounding by rapid evolution.

## Author Summary

The presence and strength of interactions between species has frequently been inferred from observational data on changes in species abundance. For example, correlated cycles in potential predator and prey species may be interpreted as evidence that the species interact, while the absence of such coupled oscillations might be interpreted as evidence for lack of interaction. Here we show that prey abundance can be decoupled from changes in predator abundance when there is genetic variability in the prey for antipredator defense traits, allowing rapid evolutionary changes in prey defense levels. It then appears that the two species are not interacting, when in fact they are. We deduce this from studies of two laboratory microcosm systems, one with algae consumed by rotifers and the other with bacteria attacked by phage. In each, when the prey vary genetically for defense traits and undefended genotypes are superior competitors, defended and undefended prey frequencies evolve in a cyclical way that is almost exactly counterbalancing, so that total prey density remains nearly constant. We show mathematically that these “cryptic cycles” occur whenever conditions are right for predator-prey cycles, when prey vary genetically for defense traits, and when prey defense against predation is effective but inexpensive to produce. Under these conditions, observations of predator and prey population dynamics cannot be trusted to be informative about the strength or even the existence of interspecific trophic links.

## Abstract 3

Contemporary rapid evolution in prey and pathogen species masks strong tropic interactions with predators and hosts. These "cryptic dynamics" reveal a need for a new approach to measuring interaction strengths in food webs.

## Introduction

Empirical and theoretical studies suggest that the structure and strength of trophic links have large influences on ecosystem attributes such as species diversity [1,2], the abundance and productivity of different trophic levels [3,4], and the stability and dynamical behavior of component populations (e.g., [5–8]).

One of the principal methods for assessing the existence and strengths of trophic interactions is an analysis of temporal patterns of change in species abundances [9]; either natural variations in abundance or transient dynamics that occur following natural or experimental disturbances of a steady state. Time-series data on changes in species abundance (and possibly on environmental covariates) are used to parameterize a multispecies dynamic model, whose parameters can then be used to calculate the various summary measures of interaction strength [10]. This approach has some advantages over strictly experimental approaches, such as species removals: in principle, the interaction strengths between all species pairs in a community can be estimated with one experiment (rather than requiring a comprehensive set of single-species removals), and estimates of direct pairwise interaction strengths are not confounded by indirect effects [9]. Recent applications include tide pool fish communities [11], freshwater plankton communities [12–14], the wolf-moose interaction in Isle Royale National Park [15,16], forest insects [17–19], and laboratory systems using microbes [20–24] and insects [25].

The fundamental premise of the system-dynamics approach to measuring interaction strengths is that the impact of one species on another is revealed by patterns of covariation in their changes of abundance through time. Although a variety of different methodologies have been used, they are all based on estimating the relationship between the abundance of one species and the rate of change in the abundance of a second, and then using some summary measure of the strength of this relationship as the estimated interaction strength.

Conclusions reached in this way may often be valid, but we have discovered that ecologically relevant conditions exist under which there is little or no relationship between the abundance of a predator and the population growth rate of its prey, and vice versa, despite the existence of what is known to be a tightly coupled interaction. Under these conditions, a strong interaction would be completely missed in an analysis based on observed changes in species abundance.

Both theoretical and experimental studies of predator–prey dynamics have assumed, explicitly or tacitly, that populations are genetically homogeneous, so that, e.g., predation does not drive nontrivial changes in prey genotype distributions even while it causes substantial changes in prey abundance. There is now considerable evidence, however, that populations may experience rapid evolutionary change contemporaneous with the ecological processes that drive changes in abundance (e.g., [26–37]). An important consequence is that if the evolving morphological, physiological, behavioral, or life-history traits influence birth or death rates, evolution may thereby radically alter population and community dynamics [38–41].

**Figure 1:**
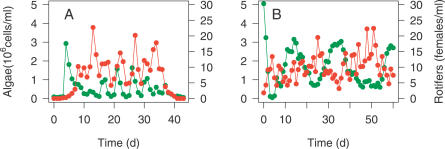
Experimentally Observed Cycles of Algae (Green) and Rotifer (Red) Populations in a Chemostat System (A) The entire initial algal population was descended from a single algal cell drawn from our stock cultures, and the predator was introduced quickly to limit algal population growth and reduce the opportunity for advantageous genotypes to arise by mutation. (B) An experiment in which the initial algal population was drawn from multiple sources and genetically variable. With prey genetic variation eliminated or greatly reduced (A), the system exhibited classical predator-prey cycles with increases in prey abundance followed by increases in predator abundance after a quarter-period lag. When the prey population is genetically heterogeneous and evolving (B), the cycles are longer and the oscillations in predator and prey abundance are almost exactly out of phase. The data are replotted from [42,57].

We report here a phenomenon that we call “cryptic dynamics,” in which the strength or even the existence of a predator–prey trophic link is masked by evolutionary dynamics. This phenomenon is a subset of the category of evolutionary cycles that we previously documented in a predator–prey interaction between a rotifer, *Brachionus calyciflorus,* and a unicellular alga, *Chlorella vulgaris,* in laboratory chemostat cultures [42]. When cultures were initiated with only a single algal genotype present, and therefore no possibility for algal evolution, we observed short-period standard predator–prey oscillations with a quarter-period phase lag between rotifer and algal peak densities (Figure 1A). This pattern was completely different, however, when multiple algal genotypes were present. The prey population cycled between dominance by genotypes defended against predation and genotypes that grew better in severely nutrient-limited conditions. Rather than short-period standard predator-prey oscillations, we observed longer-period cycles in which the rotifer and algal densities were exactly out of phase (Figure 1B) [40,42]. We have also seen qualitatively similar results in the interaction between the bacterium Escherichia coli and the lytic phage T4 that infects it [32,43]. When only sensitive hosts are present, the densities of bacteria and phage exhibit rapid high-amplitude cycles. These cycles become longer and less pronounced after phage-resistant bacteria evolve or are deliberately introduced. The striking similarity in dynamics caused by host variability in two such different systems suggests a common and fundamentally important mechanism.

Here we present experimental evidence and theoretical analyses showing that ecologically realistic conditions exist in which the prey genotype oscillations almost exactly counterbalance each other, so that total prey density remains essentially constant while predator density oscillates. When this occurs, observations of predator and prey population dynamics cannot be trusted to be informative about the strength or even the existence of interspecific trophic links.

## Results

### Unusual Predator–Prey Dynamics in Rotifer–Algal Microcosms

Our studies of rotifer–algal predator–prey cycles are carried out in single-stage chemostats, which are culture vessels with continuously pumped inflow of sterile nitrogen-limited medium and continuous outflow of medium, rotifers, and algae. Under well-understood conditions of nutrient concentration and flow rate, the tight predator–prey link between these species produces coupled population cycles [40,42,44]. Microevolution in the prey population produces the longer out-of-phase cycles described above, and alternative mechanisms (resource-dependent egg viability, stoichiometric changes in algal quality, and metabolite-dependent algal physiology) do not [45]. Theory and experimental results using identifiable algal clones show that these dynamics result from changes in algal genotype frequencies occurring in parallel with changes in algal abundance [40,42,46].

**Figure 2:**
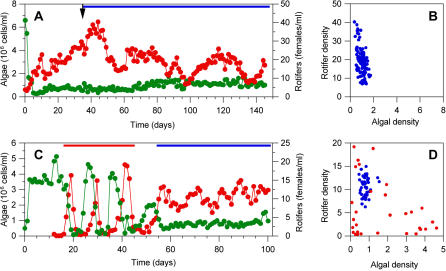
Experimentally Observed Cryptic Population Cycles in Rotifer–Alga Predator–Prey Chemostats (A) Cryptic cycles: algal density (green) stayed relatively constant, whereas rotifer density (red) fluctuated greatly. Dilution rate changed from 0.98 to 0.80 d^−1^ at day 37. (B) Densities of rotifers and algae are plotted against each other using the part of the time series indicated by the blue line in (A). (C) Transient dynamics leading to cryptic cycles. Before the rotifer population (red) was established, algal density (green) was kept high (day 0–15). Short predator–prey cycles were observed from day 16–45 (delineated by red line), presumably because a single clone dominated the algal population. Then, cryptic cycles were observed from day 55–100 (delineated by blue line). Dilution rate was 0.84 d^−1^ throughout. The estimated period of cycling, using spectral analysis for days 59–93, is 16.5 d ([52], periodicity significant at *p* < 0.001 using either Fisher's exact test or χ^2^ test). (D) Densities of rotifers and algae are plotted against each other; red and blue circles correspond to the time periods indicated by red and blue lines in (C).

We observed unexpected population dynamics, however, in several extended chemostat runs where evolutionary cycles were expected. In the first case (Figure 2A), multiple algal clones were inoculated at high density and rotifers at low density at the start of the experiment. These initial conditions should have led to long out-of-phase evolution-driven cycles, similar to those shown in Figure 1B. Instead, algal density declined initially and then stayed consistently low. At the same time, rotifer density oscillated as in a typical evolutionary cycle (especially after the drop in chemostat dilution rate on day 35 of the experiment). Based on independently measured rotifer and algal functional responses and observations of other chemostat runs, the magnitude of the changes in rotifer population size should have been more than sufficient to induce a response in algal density and, hence, in predator–prey cycling [42,44,47]. Even though we know that the rotifers and algae are bound in a tight predator–prey relationship, a plot of their densities in a predator–prey phase plane suggests instead that rotifers and algal populations are completely decoupled (Figure 2B).

In a second rotifer–algal chemostat experiment, the algal population was again initiated with multiple genotypes. In this case, however, the rotifers were not introduced until day 12, after which we observed three clear short-period cycles (Figure 2C) with the quarter-period phase delay that occurs in the absence of algal evolution (Figure 2D). In mathematical models for this system, one competitively superior clone comes to dominance during a period when, in the absence of rotifer grazing, algae at high density experience strong competition because of severe nutrient limitation. This low genetic variation would cause the system to behave initially as if no evolution were taking place, even if the frequency of a defended clone was slowly increasing during each period of high rotifer density. But once the frequency of the defended clone became high enough, the system would be expected to shift to long-period, out-of-phase evolutionary cycles. Between days 45 and 55, the dynamics did change markedly, despite there being no change in experimental conditions (Figure 2C). Consistent with expectations, the rotifers continued to oscillate with cycle period increasing from about 12 d to ∼16.5 d and with reduced amplitude. But instead of cycling out of phase with the rotifers, the algal population dropped to constant low abundance (Figure 2C). Again, the changes in rotifer density should have been sufficient to elicit fluctuations in the algal prey, but they did not, and thus dynamical evidence for trophic coupling was lacking in the predator–prey phase plane (Figure 2D; blue dots).

From the plots in Figures 2B and 2D (blue dots) alone, one would infer that B. calyciflorus and C. vulgaris did not interact in our chemostats, when in fact they are strongly and directly linked. This inconsistency led us to ask if the evolutionary cycling of genotypes within the prey population allows total prey abundance to remain essentially constant even while the predator population is cycling.

### A Mathematical Model of Cryptic Predator–Prey Dynamics

We used simple models to explore whether rapid prey evolution in response to fluctuating predation risk can produce cryptic dynamics in which prey abundance cycles with an amplitude so small that it could easily be obscured by sampling variation, environmental stochasticity, or both. Consider first the simplest case of a single predator (*y*) feeding on two clones (*x*_1_, *x*_2_) of an asexual prey species. The prey types are assumed to differ in their palatability to predators (*p*) and in a parameter *θ* affecting their birth rate:

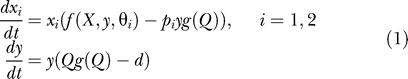
where *X* = *x*_1_ *+ x*_2_ is total prey abundance and *Q* = *p*_1_ *x*_1_ + *p*_2_ *x*_2_ is total prey quality as perceived by the predator. The function *f* represents prey births and deaths unrelated to predation, and allows for nonlethal effects of the predator (e.g., [48–51]). We assume that *f* is decreasing in *X,* nonincreasing in *y,* and increasing in a parameter *θ* affecting prey birth rate. The function *g* is the predator per capita “grazing rate,” and *p* represents prey palatability with a low-*p* clone having a lower probability of death due to predation. [Note that we use “palatability” here to signify a variety of ways that prey might be vulnerable to predator-caused mortality, not all of which have to do with true palatability (e.g., our defended algal cells survive gut passage, see below).] We number the prey types so that *p*_1_ < *p*_2_, and therefore *θ*_1_ < *θ*_2_ to represent a reproductive cost for reduced palatability. Predator population dynamics result from conversion of prey into offspring, and from a density-independent intrinsic mortality rate *d*. We also scale the model so that a unit of prey consumption yields one net predator birth.

In the Materials and Methods section, we discuss in more detail the biological assumptions underlying model (1) and their rationale. Examples of the general model (1) include the model of Abrams and Matsuda [38], in which competition between prey types is represented by Lotka-Volterra interaction terms, and a chemostat system model with two prey types competing for a limiting resource. These models are described in Protocol S1.

**Figure 3:**
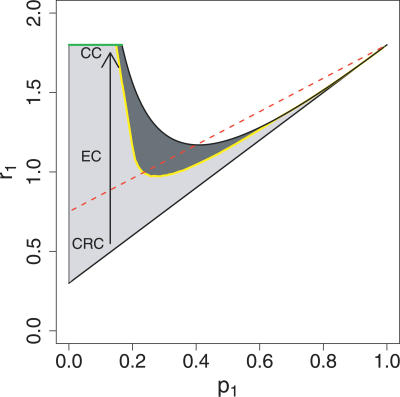
Bifurcation Diagram Showing the Parameter Regions where Evolutionary and Cryptic Cycles Result from Rapid Prey Evolution in Model (1) This diagram is drawn for the Abrams-Matsuda [38] model (Equation S1.1 in Protocol S1) in which *r* plays the role of the birthrate parameter θ. Parameters for this plot are *d* = *p*_2_ = 1, *G* = 2, *K* = 0.3, and *r*_2_ = 1.8, which gives a consumer-resource limit cycle when the defended prey (type 1) is absent. The region shaded in gray indicates values of defended prey parameters such that there is an equilibrium with both prey types and the predator at positive density; dark (respectively light) gray shading indicates that the equilibrium is stable (respectively unstable). The red dashed line shows the minimum value of *r*_1_ for which the defended prey can invade the (predator + vulnerable prey) limit cycle [78]. If defense is effective (*p*_1_ ≪ 1) but costly (*r*_1_ ≪ *r*_2_), the system has consumer-resource cycles (CRCs) with the defended prey absent. As the cost of defense is decreased (so that *r*_1_ increases as indicated by the arrow), the defended prey becomes able to invade. This invasion results in evolutionary cycles (ECs) with the predator and total prey densities cycling out of phase, rather than the quarter-phase lag in ordinary consumer-resource cycles. As the limit *r*_1_ = *r*_2_ is approached, the evolutionary cycles become cryptic cycles (CCs), meaning that total prey density is nearly constant. The asymptotic analysis in the text shows that cryptic cycles always occur for parameters near the intersection of the two bifurcation curves (green and yellow): disappearance of the coexistence equilibrium at *r*_1_ = *r*_2_ (green) and the transition from stability to cycling (yellow).

“Cryptic” cycles in model (1) are a limiting case of the evolutionary cycles described above. A general analysis of model (1) [52] shows that evolutionary cycles occur when: (a) defense is effective but not too costly (*p*_1_ ≪ *p*_2_ but *θ*_1_ ≈ *θ*_2_), and (b) the coexistence steady state for model (1), at which both prey types coexist with the predator, is locally unstable (see Figure 3). A coexistence steady state always exists for *p*_1_ ≪ *p*_2_ and *θ*_1_ ≈ *θ*_2_, and it is always a spiral point but may be stable or unstable.

In both the Abrams-Matsuda and our chemostat models, these conditions are all satisfied for *p*_1_ ≈ 0 when the reduced system consisting of the predator and the more vulnerable prey exhibits consumer-resource cycles [52]. However, as *p*_1_ increases with *θ*_1_ close to *θ*_2_, the coexistence steady state in model (1) always becomes locally stable (before eventually disappearing with the vulnerable prey type dropping out due to indirect “apparent” competition with the defended prey). These qualitative behaviors are not dependent on either parameter values or the functional forms of *f* and *g* in model (1).

The mechanism for cryptic cycles is density compensation, by which we mean that the two prey types cycle out of phase with each other in such a way that their total abundance remains nearly constant. Evolutionary cycles in model (1) always develop this character as the cost of defense becomes small (*θ*_1_→ *θ*_2_). An asymptotic analysis treating ɛ = *θ*_2_ − *θ*_1_ as a small parameter [52] shows that the dominant eigenvector of the Jacobian at the coexistence equilibrium (which gives the linear approximation to small-amplitude cycles) is

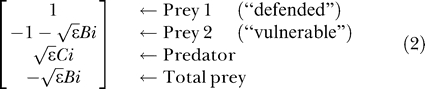
where 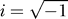 and *B* and *C* are positive constants. The angle in the complex plane between the eigenvector components for different state variables corresponds to the phase lag between their cycles, e.g., the approximately 180° angle between Prey 1 and Prey 2 corresponds to half of a cycle period. The entries in (2) therefore imply that the following: (a) the two prey types are almost exactly out of phase with each other; (b) the predator and total prey are exactly out of phase with each other; (c) the cycles of the predator and the vulnerable prey type exhibit the quarter-period lag typical of classical consumer-resource cycles.

A dimensionless measure of each population's variability is given by the ratio between its cycle amplitude and its abundance at the coexistence equilibrium (which lies approximately at the center of near-equilibrium cycles). Cycle amplitudes are proportional to the magnitudes of the eigenvector components in (2). As the cost of defense becomes very small (ɛ → 0), the equilibrium total prey abundance approaches a finite limit while the predator equilibrium is order ɛ (e.g., a 50% reduction in ɛ leads to a roughly 50% reduction in the predator equilibrium density). Therefore, relative to the evolutionary variability in the prey (i.e., the relative proportion of the two types), the variability of total prey abundance is order 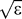 smaller, whereas the predator variability is order 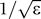 larger. In addition, the cycle period converges to infinity as ɛ → 0 [52]. Prey evolution is driven by occasional predator outbreaks, but prey density remains nearly constant because the consumption of the vulnerable prey is almost exactly balanced by growth of the better-defended prey when they are released from competition with the vulnerable type.

**Figure 4:**
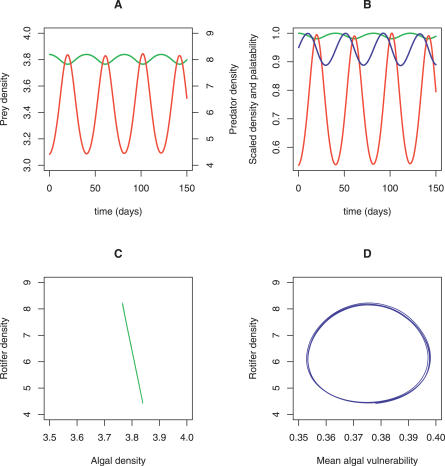
Numerical Solutions of the Chemostat Model Showing Cryptic Cycles Resulting from Rapid Prey Evolution (A) Total prey (green) and predator (red) densities. (B) Mean algal palatability to predators (blue), and total prey (green) and predator (red) densities expressed in dimensionless units by scaling relative to their maximum values over the time interval plotted, in order to show the relative scales of variability. As predicted by the asymptotic analysis, there are substantial (nearly 2-fold) proportional oscillations in predator density, moderate oscillations in prey palatability reflecting prey evolution, and very small oscillations in total prey density. (C) Phase-plane plot of predator and total prey densities. Because of the predator-prey phase relationship during evolution-driven cycles, the phase plot lies on a very narrow and nearly vertical band (Figure 2B). (D) Phase-plane plot of predator density and mean prey palatability, showing the “predator-trait” limit cycle. Predator and vulnerable prey parameters for this plot are the values estimated for our rotifer-algal chemostat system [42]; we set the dilution rate to δ = 1, and gave the defended prey type *p*_1_ = 0.1 (relative to *p*_2_ = 1 for the vulnerable prey) and a half-saturation constant for nutrient uptake 5% higher than that of vulnerable prey. The full rotifer–alga model includes predator age-structure and predator mortality within the chemostat, which are not in model (1); to eliminate these we set *m* = λ = 0 in the full model. However, the behavior of the model is very similar if the estimated values (*m* = 0.055, λ = 0.4) are used, except that cycles then occur for a wider range of *p*_1_ values.

A caveat to these conclusions is that they are based on the Jacobian at the unstable coexistence equilibrium, so they are only guaranteed to be a good approximation if cycles stay fairly close to the equilibrium. That is, our analysis guarantees the occurrence of cryptic cycles only for parameter values near the intersection of the green and yellow bifurcation curves in Figure 3. However, model simulations show that the cycles can remain cryptic—in the sense that the cycle amplitude of total prey density is very low—even far from the transition between cycling and stability. Figure 4 shows numerical solutions of the chemostat model, with parameters estimated for our algae-rotifer experimental system [42]. In these simulations, we set *p*_1_ = 0.25, *p*_2_ = 1, and δ = 1, and the defended prey had a half-saturation constant for nutrient uptake 5% higher than that of vulnerable prey; with these parameters, the transition between stability and cycling occurs at *p*_1_ ≈ 0.4. Plotting the predator and total prey densities and the mean prey palatability relative to their temporal averages, the scales of variability match the near-equilibrium analysis even though the predator density varies by nearly a factor of 2: predator density variability > prey evolution > total prey variability. Predator and total prey densities are exactly out of phase, whereas there is a quarter-period lag between predator density and the mean prey palatability. So instead of a predator–prey cycle, we observe a “predator–trait cycle,” in which the “resource” being “consumed” is the mean palatability of the prey population.

**Figure 5:**
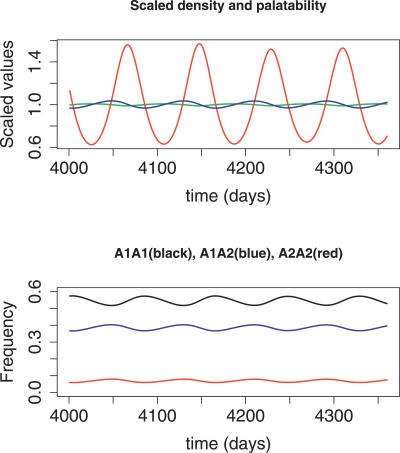
Numerical Solutions of the Chemostat Model with Diploid, Sexually Reproducing Prey Parameters for this were otherwise the same as in Figure 4, with the two prey homozygotes having the palatability and half-saturation values of the two prey clones in Figure 4. The prey heterozygote has the average of the homozygote half-saturation values, but the heterozygote palatability was closer to that of the palatable homozygote, *p*_12_ = 0.3*p*_11_ + 0.7*p*_22_. The upper panel shows (as in Figure 4) the total prey density (green), predator density (red), and mean prey palatability (blue), put into dimensionless units by scaling each variable relative to its temporal average over the time period plotted. The lower panel shows the frequencies of the three genotypes (black = *A*_1_ *A*_1_, blue = *A*_1_ *A*_2_, red = *A*_2_ *A*_2_).

The algae and rotifers in our chemostat system are both obligately asexual, and our model up to this point is structured in accordance with this fact. To show that the phenomenon of cryptic cycles is not limited to asexual species, we consider a simple model with a sexually reproducing diploid prey species. For simplicity, we assume that defense is determined by a single locus with alleles *A*_1_, *A*_2_ such that *A*_1_ *A*_1_ genotypes have palatability *p*_11_ ≪ 1, *A*_2_ *A*_2_ genotypes have palatability *p*_22_ = 1, and *A*_1_ *A*_2_ genotypes are intermediate. Model equations are given in Protocol S1. Figure 5 shows that a chemostat model with sexual reproduction can still exhibit cycles in which total prey abundance remains nearly constant. However, the model's behavior is sensitive to the assumed tradeoff curve between predator defense and nutrient uptake ability (the asexual model is much less sensitive to the shape of the tradeoff curve because only extreme types coexist [*p*_1_ ≈ 0, *p*_2_ ≈ 1]). In Figure 5, the heterozygote has half the cost of the defended genotype, but gains only 30% of the benefit, *p*_12_ = 0.3*p*_11_ + 0.7*p*_22_. If *p*_12_ is instead closer to *p*_11_, which gives more of an advantage to heterozygotes, the cycles of predator abundance and prey genotype frequencies are much smaller.

### Cryptic Population Cycles in Phage–Bacterial Microcosms

The data from rotifer–algal chemostats led us to explore the possibility that rapid prey evolution could underlie our experimental observations of predator cycling without an accompanying response in total prey density, and mathematical modeling demonstrates that this is a plausible explanation. However, for the rotifer–algal experiments, we do not have direct evidence of changes in algal genotype frequencies that would confirm this interpretation. There is, however, another predator–prey chemostat system that provides direct evidence of cryptic dynamics: a bacterial prey, *E. coli,* attacked by lytic bacteriophage T4 [32,43].

**Figure 6:**
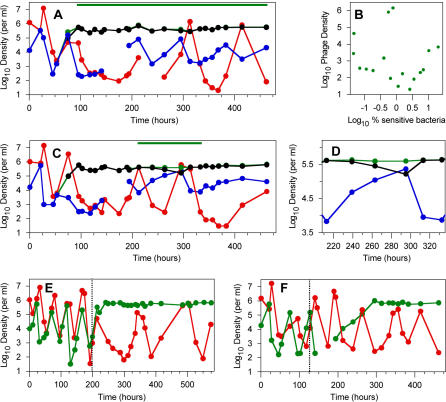
Experimentally Observed Cryptic Cycles in Bacteria–Phage Chemostats (A) Results from [32] showing densities of phage (red), sensitive bacteria (blue), resistant bacteria (black), and total bacteria (green, mostly hidden by black). Resistant bacteria with a neutral genetic marker were introduced ≈75 h after the experiment was initiated with phage and sensitive bacteria. (B) The relationship between phage density and the fraction of sensitive bacteria, plotted for the time period indicated by the dark green line at the top of (A), which omits the period before the introduction and transient increase of the resistant strain. (C) Results [32] plotted as in (A). (D) A “blowup” of the time period indicated by the dark green line at the top of (C), when the sensitive strain increased greatly during a period of low phage density. These data show density compensation, with total bacterial density remaining nearly constant due to opposing changes in the abundance of the two bacterial strains. (E and F) Two experiments where the spontaneous appearance of a second, resistant, bacterial type led to a change in dynamics: stabilization of total bacteria, and longer cycle periods in the phage. In both panels E and F, the dashed vertical lines show when the resistant strain was first detected; because the resistant type did not have a neutral marker, the separate abundances of resistant and sensitive bacteria could not be tracked accurately once the resistant strain became numerically dominant. The data in panel E are from ([43], Figure 3B), data in F are from [32] .

Figure 6 shows results from two experimental runs that were initiated with phage and a bacterial strain that was susceptible to phage attack. After ≈75 h, a second bacterial strain, resistant to phage attack, was introduced. Critical for our purposes here, these resistant bacteria carried a neutral marker that made it possible to reliably estimate the separate densities of the resistant and sensitive strains (see Materials and Methods). In both cases (Figure 6A and 6C), there were 1–2 population cycles of the phage and sensitive bacteria; addition of the resistant strain was followed by stabilization of total bacterial density (within experimental error) but continued cycling of phage density. The fraction of the susceptible genotype in the total bacteria population clearly showed evolutionary cycles in concert with cycles in bacteriophage density, as our model predicts. Changes in the fraction of sensitive bacteria produced oscillations in phage density over five orders of magnitude, so the tight coupling between the populations is revealed by a phase-plane plot of bacteriophage density against the fraction of susceptible bacteria (Figure 6B).

These experiments thus confirm that cryptic cycles were produced by genetic diversity and rapid evolutionary dynamics in the host population. In Figure 6A, the density of the sensitive strain becomes so low relative to the total bacterial density (note that density is plotted on a log scale) that we have no evidence either for or against the theoretical prediction of density compensation between the sensitive and resistant strains. The changes in sensitive-strain density were swamped by the uncertainty in resistant-strain density (as estimated from the unexplained between-sample variability). Several other runs with the same experimental design ([32,43] and unpublished data) exhibited the same features: total bacterial density ceased to cycle (within experimental error) because only a small fraction of the total population was interacting with the phage. However, when the sensitive-strain density did increase appreciably, during a period of low phage density (Figure 6C and 6D), we observe nearly exact density compensation between the two host strains, as predicted.

Figure 6E and 6F shows results from two other experiments in which a resistant bacterial strain arose spontaneously by mutation within the experimental cultures. Because this strain lacked the neutral marker carried by deliberately introduced resistant strains, we know when the resistant strain arose but do not have reliable separate estimates of sensitive- and resistant-strain densities (see Materials and Methods). However, because the resistant strain arose later in these experiments than in those where the resistant type was deliberately added, they provide the clearest evidence for the predicted changes in population-level dynamics. Once the resistant strain arose and achieved high density, the total host density stabilized while the density of the phage continued to cycle, but with the markedly longer cycle period characteristic of evolution-driven cycles.

## Discussion

We observed unexpected dynamics in our predator–prey microcosms: predator density oscillated while prey density remained nearly constant. Our theoretical results suggest that rapid prey evolution can explain this pattern. A role for rapid contemporary evolution in population dynamics has been long hypothesized [26,27,30,31,38,41,53–56] and, more recently, has been demonstrated in both laboratory (e.g.,[28,32,38,42,46,57] ) and field studies (e.g., [58–61]). In our chemostat systems, defended prey genotypes increase in frequency when predation is severe, and undefended but competitively superior genotypes become dominant when predation pressure is absent or low. We have shown here that ecologically relevant conditions exist in which the oscillations in prey genotype abundances are almost exactly compensatory, so that total prey density stays essentially constant. Even subtle changes in prey gene frequency can produce substantial responses in predator density (Figures 4–6). We call this pattern “cryptic dynamics,” because data on predator and prey population densities alone would lead to the incorrect conclusion that no interaction was present. We infer for the rotifer–algal system and we have shown directly for the phage-bacterial system that cryptic dynamics underlie the experimental results shown in Figures 2 and 6.

Our theoretical analyses show that cryptic dynamics are expected when a predator–prey limit cycle would occur in the absence of evolution, when a tradeoff exists between the prey genotypes in which the better defended genotypes are poorer competitors for limiting nutrients and vice versa, and when the cost of defense is fairly low; if the cost is too high, then noncryptic evolutionary cycles occur [42]. For the results reported here, the chemostats were run under conditions known to produce limit cycles [43,44]. Fitness tradeoffs have been documented for both C. vulgaris [47] and E. coli [28,43,62], and we infer for the algae and bacteria involved in the interactions in Figures 2 and 6 that the cost of defense was low enough to produce cryptic dynamics.

These cryptic dynamics can be thought of as part of a larger phenomenon in which the strengths of ecological interactions at one level of organization are obscured by compensatory interactions at a lower level. In food webs, the degree to which higher trophic levels control the density or productivity of lower levels through trophic cascades [3,63] varies substantially in magnitude and persistence [64–66]. The explanation in at least one freshwater pelagic ecosystem is the presence of a cryptic trophic cascade in which the phytoplankton community responds to variation in grazer abundance through changes in the relative abundance of species of varying palatability and competitive ability, whereas the total abundance of the trophic level remains relatively constant [67]. Such dampened or wholly cryptic trophic cascades are expected when heterogeneity exists within a trophic level, thereby leading to compensatory responses by component species [64,67,68]. Our results show that such compensatory responses can also occur within a single population through life-history tradeoffs among genotypes.

A well-established method for determining the strengths of trophic interactions in food webs in the wild is to analyze statistical relationships between the abundance of potential predators and that of their presumed prey. The potential for the kind of cryptic dynamics that we have documented here means, however, that there are circumstances in which the absence of such statistical relationships cannot be reliably taken as evidence for the absence of important biological interactions. Even if a relationship is found because the dynamics are not completely cryptic, the strength of the interaction cannot be reliably inferred when the prey evolve quickly in response to changes in predator abundance.

If prey evolution can obscure predator–prey coupling in studies of variation in natural abundances, what about in enclosure experiments in which predator density is manipulated and the strength of the interaction is inferred from the changes in prey abundance? One common experimental approach is to construct enclosures, which either surround portions of natural communities or in which artificial communities are established, and then to supplement or remove a species whose impact one wishes to measure. In a PRESS experimental design, the density of one species is altered and then held constant at the new level, and the subsequent dynamics of predator and prey density are followed through time.

**Figure 7:**
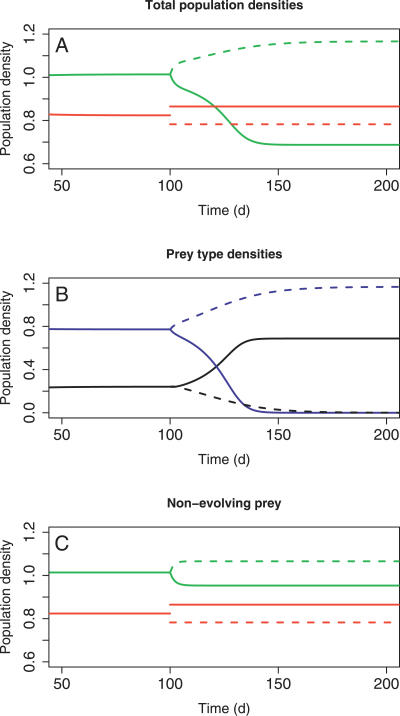
Simulation of a Predator–PRESS Experiment. We used the Abrams-Matsuda [38] model for this simulation. From time *t* = 0 to *t* = 100 the populations are unperturbed and converge to a stable equilibrium with both prey types present. At *t* = 100 the predator population is increased (solid curves) or decreased (dashed curves) and thereafter held constant. (A) The predator (red) and prey (green) total population densities. (B) The separate abundances of the defended (black) and vulnerable (blue) prey genotypes. Decreased predator density gives an advantage to the vulnerable prey type, while increased predator density gives the advantage to the initially rare defended prey type, leading to a large change in total prey density. (C) As in (A), but without prey evolution, i.e., the prey population consisted of a single type having the average characteristics (palatability and birth rate parameter) of the overall steady-state prey population in (A).

We evaluated the effect of rapid contemporary evolution on the dynamics of a typical enclosure PRESS experiment using the Abrams-Matsuda [38] model (Protocol S2). Interestingly, the predicted effect of prey genetic variation in predator PRESS experiments is typically the amplification of the effect of changes in predator abundance. Even a small change in predator density sets off a cryptic evolutionary “cascade” in which one prey type excludes the other, potentially resulting in a very large change in total prey density, much larger than would have occurred with a single prey type (Figure 7). The long-term effect of the predator manipulation then depends on the direction of the change in predator density, but not on the amount of change. The interaction strength would be drastically over-estimated in cases where a small predator manipulation yielded a large response in prey density. However, some amplification will always occur, so long as the more predation-resistant prey are less effective at resource acquisition: predator addition (reduction) tips the balance in favor of resistant (competitive) prey, and that change in prey-type frequency will decrease (increase) prey abundance independent of the change in predation rate.

In a typical predator PULSE experiment, by contrast, predator density is quickly increased or decreased, but the predator and prey populations are then allowed to change without further interventions. The effect of prey evolution on such experiments depends on the relative time scales of predator and prey population change (i.e., on their relative generation times). If the predator's generation time is much longer than that of the prey, a predator PULSE is effectively a PRESS whose effects could be drastically amplified by prey evolution. However, if the generation times are comparable, then prey evolution can alter the long-term system dynamics following a PULSE (e.g., changing the return to equilibrium from monotonic to oscillatory) but the initial response may not be greatly affected because the predator's return to (or overshoot of) its pre-PULSE steady state causes the prey-type frequencies to swing back towards (or overshoot) their pre-PULSE values. Despite concerted effort using both the chemostat and Abrams-Matsuda models, when predator and prey generation times are roughly equal we did not find parameters where prey evolution changed the initial response (e.g., the first minimum in prey abundance following a predator increase) by much more than a factor of 2. Thus, it appears that a predator PULSE experiment may give reasonably accurate estimates of interaction strength in the presence of rapid prey evolution if the predator and prey have similar generation times.

### Conclusions

Cryptic population dynamics take place when the nature of an ecological interaction is obscured by rapid evolution in one or more of the interacting species. Here we have provided experimental examples in which cycling by a resource species was effectively eliminated through compensatory changes in the frequencies of prey genotypes that differed in their vulnerability to a consumer. Using a mathematical model, we have established the conditions necessary for these cryptic cycles: (1) predator–prey cycles would occur between the consumer and the more vulnerable genotype of the resource species if that genotype were the only one present, (2) the less vulnerable of the resource genotypes has an effective defense against the consumer, and (3) the cost of defense is fairly low in terms of ability to compete for limiting resources.

Empirical studies suggest that these requirements often hold in natural systems. Cyclic population dynamics are widespread across all major animal taxa and biomes, occurring in roughly 30% of the available long-term data sets on population variability [69]. Fitness costs for defensive compounds and structures have often proved difficult to demonstrate, and in many cases, either no tradeoff or only a weak tradeoff was found (e.g., [47,70–76]) . Therefore, efforts to establish the nature and strength of interactions in ecological communities that fail to consider the potential for evolution (which is to say virtually all efforts to date) run a risk of being incorrect. Because essentially all natural populations have heritable variation for ecologically important traits, and the number of examples of rapid contemporary evolution is large and growing, ignoring the potential for evolution to affect measurements of species interaction strengths becomes untenable.

Although our microcosms are extremely simple systems, they mimic the consumer–resource interactions occurring in natural systems. Our rotifer–algal interaction is an herbivore consuming a primary producer (though we call it “predator–prey” because each algal cell is consumed whole), and our phage–bacterial interaction can be considered as either predator–prey or host–parasitoid (in which successful infections are lethal). Parasite–host dynamics can be significantly altered by contemporary evolution [30,77], so epidemiological predictions of disease outbreaks may well need to take account of evolution.

The focus here has been on the ecological consequences of evolution in prey populations, in particular showing how such evolution can obscure the coupling between predator and prey dynamics. Of course the processes and patterns we have described in prey populations can also have further evolutionary consequences. We suggest two. Cryptic evolutionary cycles result in the maintenance of non-neutral genetic variation in isolated populations at equilibrium abundance. In nature, variation harbored by this mechanism would be grist for the mill of rapid adaptive evolutionary response to environmental change of the kind reported with increasing frequency [26–37,41]. Second, predators might evolve that partially or completely overcome the defenses of the more resistant prey type. This outcome could, in turn, lead to cycles of predator–prey coevolution, perhaps leading to adaptive radiation in the prey, the predator, or both populations, depending on the existence and pattern of tradeoffs between competitive ability and resistance in the prey population, and between growth rates on different prey types in the predator population. However, the particular evolutionary path will depend on details of any given predator–prey interaction [28,39]. We have, in fact, sometimes observed evolutionary changes in rotifers (but for a character unrelated to diet), and certain phage species can sometimes evolve to overcome bacterial resistance (but not the phage T4 we used) [28,40]. If a predator eventually evolves the renewed ability to consume the defended prey, then traditional predator–prey cycles might reappear. However, it has not been necessary to incorporate evolution of the predator in order to explain the cryptic dynamics that we observe in both of these systems. More generally, it is little consolation that the traditional predator–prey coupling might (or might not) be evident depending on the patterns of genetic variation in one or both populations, because so little is usually known about that variation.

We have shown that the coupling of ecological and evolutionary dynamics can have unexpected consequences even in the simplest possible ecological community. We expect that further surprises will be found as the effects of evolution are traced in more complex communities and ecosystems. If rapid evolution is pervasive, then all of ecological theory needs to be re-examined to take into account the fact that changes in distribution and abundance are likely to be accompanied by evolutionary dynamics that, in turn, alter the very changes in distribution and abundance that we are striving to understand. It is a daunting and exciting prospect.

## Materials and Methods

### Rotifer–algal chemostats.

Our rotifer–algal chemostat system has been described in detail elsewhere [40,44,57] so we give here only an outline. We established stock cultures of C. vulgaris (UTEX C. vulgaris culture no. 26; http://www.utex.org) and Brachionus calyciflorus (taken originally from the harbor at Milwaukee, Wisconsin, United States, and provided by M. Boraas). We established that our algal stock culture is genetically variable for ecologically relevant traits, because clonal populations that were derived from it exhibit heritable phenotypic changes in response to selection [47]. Defended cells survive gut passage when consumed by rotifers, but have a reduced growth rate at low nutrient concentrations [46,47].

The experiments reported here used 380-ml, single-stage chemostats to culture these organisms in sterilized medium with nitrogen (in the form of nitrate) as the limiting nutrient, under constant light (120 μE m^−2^ s^−1^) and temperature (25 °C). We set the dilution rate (0.80–0.98 d^−1^) and nutrient concentration (80 μmol/l nitrate) to give population cycles based on results in [42,44]. Organisms were sampled daily through ports near the bottom and top of each chemostat. Rotifers were counted under a dissecting microscope and algae were counted with a particle counter (CASY 1, Schärfe; http://www.casy-technology.com). Organism abundance data are presented as means of duplicate samples.

For our work, it is important to know that the chemostats were not accidentally contaminated with other species, so that we can be sure of a strong direct trophic link between algae and rotifers. No other species were observed during visual counting of rotifers. The size distribution of suspended particles (obtained from the particle counter) showed a clear single peak corresponding to algal cell size. This suggests the absence of other organisms or bacteria, which produce a peak at smaller size than our algae if they are present (T. Yoshida, unpublished data). Also, nitrate concentrations were consistently very low (0.24–0.56 μmole l^−1^) compared with fresh medium (80 μmole l^−1^), while algal density stayed unchanged and rotifer predator density fluctuated (T. Yoshida, unpublished data), suggesting that the limiting resource was not being captured by some other species that the rotifers could then consume. Thus, it is unlikely that any other species were present in sufficient numbers to affect the population dynamics of the rotifer-algal system.

### Phage–bacterial chemostats.

The bacteria–phage chemostat system has been described in detail previously [32,43] so we give here only an outline. The experiments used E. coli and bacteriophage T4 cultured in single-stage chemostats, with limiting glucose supplied at 0.5 or 0.1 mg/l of fresh culture medium. Chemostats were maintained at a volume of 30 ml, temperature 37 °C, and dilution rate 0.2/h. Experimental runs were inoculated with phage T4 and *E. coli* B strain REL607, which is susceptible to attack by T4. T4-resistant mutants were either inoculated deliberately at ≈75 h into the run (E. coli B strain REL6584), or arose spontaneously in control chemostats. All T4-resistant mutants in this E. coli strain achieve resistance through the loss of particular moieties on the lipopolysaccharide core surface receptor to which T4 binds to initiate infection [62]. This loss confers complete invulnerability to attack by T4, at the cost of a competitive disadvantage under glucose-limited conditions when phage are absent [28,32,43,62].

Total bacteria and phage densities were estimated twice daily by dilution and plating. REL607 density was estimated on agar plates containing arabinose as a sole carbon source; REL6584 is unable to use arabinose, so this medium allows growth of REL607 but not REL6584 (the inability to use arabinose is selectively neutral in the culture medium used for all chemostat runs). Phage density was estimated by plating on a lawn of REL607. REL6584 density was estimated by mixing a second bacterial sample with a concentrated T4 lysate (which kills the sensitive strain, REL607) and then plating on a lawn of glucose medium. See [32,43] for details of these procedures. Spontaneously arising T4-resistant mutants did not carry the marker (inability to use arabinose) and so could not be counted separately by these methods. When resistant mutants are rare, plating after mixture with T4 lysate gives an estimate of the resistant strain, while plating on arabinose gives an estimate of total bacterial density. But once the resistant strain becomes numerically dominant, sampling variability is too high for the sensitive-strain density to be estimated by the difference between total and resistant strain density, as both plating methods are really estimating total bacterial density.

### Assumptions of the general model (1).

A key biological assumption in model (1) is total niche overlap between the prey types, which is reflected in *f* being a function of total prey density *X*. This assumption seems reasonable for within-species heritable variation, especially when the prey's resource base is homogeneous. Another important biological assumption is that the function *g*, which can be thought of as the predator attack rate, depends on *Q* rather than on *X*. This assumption can be justified mechanistically in at least two different situations:

First, suppose that the mechanism of prey defense is crypsis, with *p* representing the probability that a prey individual is detected by a predator searching the area containing that individual. Then the instantaneous capture rate is the same as if the prey abundances were *p*_1_ *x*_1_ and *p*_2_ *x*_2_, but each predator can detect all prey within its search area. This leads to the predation rates in model (1), with *xg(x)* being the capture rate by one predator at density *x* of visible prey.

Second, and more relevant to our rotifer-algal chemostat system, suppose that the predator is an aquatic filter-feeder and defended prey have a higher probability of passing through the predator gut undigested and unscathed, as in our rotifer-alga experimental microcosms [46]. If the predator adjusts the volume of water it filters per unit time (clearance rate) in response to the total number of digestible prey *Q,* then the per-predator rate of prey consumption will be of the form *Qg(Q)*, as assumed in model (1). Although we have no direct evidence on clearance rates in our microcosms to support this assumption, models with *Q*-dependent clearance rate were more successful at quantitatively matching experimental data on cycles in predator and total prey abundance [42] than were models with *X*-dependent clearance rate, and the grazer population growth rate is *Q*-dependent [47].

## Supporting Information

Protocol S1 Examples of the General Model (1) (147 KB DOC)

Protocol S2 Analysis of PRESS Experiments (60 KB DOC)
