# Supplementary material for: Cryptic Population Dynamics: Rapid Evolution Masks Trophic Interactions
Source: PLoS Biol. 2007 Sep 4;5(9):e235. doi: 10.1371/journal.pbio.0050235 (PMC1964773; doi:10.1371/journal.pbio.0050235)
Supplement: Supplementary file 1 [file pbio.0050235.sd001.doc]

## **Protocol S1: Examples of the General Model (1)**

## Abrams-Matsuda Model

Abrams and Matsuda [1] modeled a single predator feeding on two prey types by generalizing the classical Rosenzweig-MacArthur model. Competition, both within and between prey types, is modeled using Lotka-Volterra interaction terms. Predation is modeled by a type-II functional response, with the prey differing in their palatability. Here we add the assumption that the prey are identical apart from their palatability and its cost in terms of reduced net birth rate, so the competitive effect experienced by each prey type is a function of the total prey density. After some rescaling of state variables the model equations are

$$\begin{matrix} \frac{dx_{i}}{dt}=x_{i}\left(r_{i}-X-p_{i}\frac{gy}{K+Q}\right), i=1,2 \\ \frac{dy}{dt}=y\left(\frac{gQ}{K+Q}-d\right) \end{matrix}$$

The model can be rescaled further, without loss of generality, so that $p_{2}=d=1$.

Besides simplicity, this model has the advantage that the conditions for prey coexistence and cycling have been thoroughly studied (e.g., [1,2,3]). With only prey 2 present and $p_{2}=1$, reduces to a classical Rosenzweig-MacArthur model. There is a steady state at which predator and prey coexist so long as $r_{2}>\bar{Q}\equiv dK/(g-d)$and otherwise the system collapses to the prey alone; $\bar{Q}$ is the value of *Q* at which $dy/dt=0$. The (predator + prey 2) coexistence steady state is locally stable for $r_{2}<(g+d)K/(g-d)$ and unstable (leading to a predator-prey limit cycle) if the opposite inequality holds. For $r_{2}>\bar{Q}$ direct calculations show that there is an equilibrium for with the predator and both prey types at positive density for any $0\leq p_{1}<1$ and

$p_{1}r_{2}+(1-p_{1})\frac{dK}{(g-d)}\leq r_{1}\leq\min\left(r_{2},p_{1}r_{2}+\frac{1-p_{1}}{p_{1}}\frac{dK}{(g-d)}\right)$;

this is the gray shaded region drawn in Figure 3. The two sections of the upper limit meet at the point $p_{1}=p*=\bar{Q}/r_{2},r_{1}=r_{2}$. It has been shown [4] that the coexistence equilibrium is always locally stable for $(p_{1},r_{1})$ values near $(p*,r_{2})$.

## Chemostat models

The standard chemostat model (e.g.,[5]) assumes that population growth is limited by a single critical nutrient. Growth medium with a constant concentration of the limiting nutrient flows into the culture vessel at a constant rate. The system is assumed to be well-mixed, and the volume of growth medium in the vessel is held constant by continuous outflow at the same rate as the inflow. After scaling to dimensionless units, the standard model with one predator and two asexually reproducing prey is

$$\begin{matrix} \frac{dS}{dt}=\delta(1-S)-x_{1}f_{1}(S)-x_{2}f_{2}(S) \\ \frac{dx_{i}}{dt}=x_{i}\left(f_{i}(S)-\delta-p_{i}\frac{gy}{K+Q}\right) \\ \frac{dy}{dt}=y\left(\frac{gQ}{K+Q}-\delta\right) \end{matrix}$$

where *S* is the scaled concentration of the critical nutrient and *δ* is the *dilution rate*, the fraction of culture medium replaced per unit time. In this scaling, all state variables are measured relative to the steady-state amount of limiting nutrient in the culture vessel. Without loss of generality, we rescale time so that *δ*=1*.* The total nutrient $\Sigma=S+x_{1}+x_{2}+y$ satisfies $d\Sigma/dt=1-\Sigma$, so the system converges onto the plane $\Sigma\equiv1$. We can therefore drop *S* from by substituting $S=1-x_{1}-x_{2}-y$ into second and third lines of , giving a 3-dimensional model of the form (1).

To complete the model we need to specify the nutrient uptake functions $f_{1},f_{2}$. For the genetic variability arising spontaneously in our rotifer-alga chemostat systems. prey genotypes with better defense against predation had lower population growth rates, but only when nutrients were very scarce [6]. We therefore assume that the prey types differ only in their half-saturation constants for a Monod model of nutrient uptake:

$f_{i}(S)=\frac{\rho S}{k_{i}+S}$.

The scaled model is then

$\begin{matrix} \frac{dx_{i} }{dt}=x_{i}\left(\frac{\rho(1-X-y)}{k_{i}+(1-X-y)}-1-p_{i}\frac{gy}{K+Q}\right) \\ \frac{dy}{dt}=y\left(\frac{gQ}{K+Q}-1\right) \end{matrix}$.

The qualitative behavior of is very similar to that of the Abrams-Matsuda model (existence and qualitative shape of the coexistence region, occurrence of evolutionary cycles, etc.), but explicit calculations such as are not possible [4].

To model a chemostat with sexually reproducing prey, let $b_{ij}$ and $d_{ij}$ denote the instantaneous birth and death rates of the prey genotypes. We assume that the birth rates $b_{ij}$ represent the rate at which individuals produce pairs of gametes, and that new offspring are formed by random combination of gametes. The birth rate of genotype $ij$ is and the death rate is the sum of predation and washout rates, $d_{ij}=1+p_{ij}\frac{gy}{K+Q}$ with $Q=p_{11}x_{11}+p_{12}x_{12}+p_{13}x_{13}$. The total instantaneous rate of gamete-pair production is then

$$B(t)=b_{11}(t)x_{11}(t)+b_{12}(t)x_{12}(t)+b_{22}(t)x_{22}(t)$$

where $x_{ij}$is the abundance of genotype *ij*, and the fractions of $A_{1}$ and $A_{2}$ gametes are

The dynamics of genotype abundance are then

$$\begin{matrix} \frac{dx_{11}}{dt}=\varphi_{1}^{2}B-d_{11}x_{11} \\ \frac{dx_{12}}{dt}=2\varphi_{1}\varphi_{2}B-d_{12}x_{12} . \\ \frac{dx_{22}}{dt}=\varphi_{2}^{2}B-d_{22}x_{22} \end{matrix}$$

The differential equations for clone abundances, $x_{1}$ and $x_{2}$ in , are then replaced by the three equations in for $x_{11},x_{12}$ and $x_{22}$.

**References**

1. Abrams PA, Matsuda H (1997) Prey adaptation as a cause of predator-prey cycles. Evolution 51: 1742-1750.

2. Abrams PA, Kawecki TJ (1999) Adaptive host preference and the dynamics of host-parasitoid interactions. Theoretical Population Biology 56: 307-324.

3. Kretzschmar M, Nisbet RM, McCauley E (1993) A predator-prey model for zooplankton grazing on competing algal populations. Theoretical Population Biology 44: 32-66.

4. Jones LE, Ellner SP (2007) Effects of rapid prey evolution on predator-prey cycles. Journal of Mathematical Biology: in press.

5. Smith HR, Waltman P (1995) The Theory of The Chemostat: Dynamics of Microbial Competition. Cambridge: Cambridge University Press. 313 p.

6. Yoshida T, Hairston NG, Ellner SP (2004) Evolutionary trade-off between defence against grazing and competitive ability in a simple unicellular alga, *Chlorella vulgaris*. Proceedings of the Royal Society of London Series B-Biological Sciences 271: 1947-1953.
