# Supplementary material for: Cryptic Population Dynamics: Rapid Evolution Masks Trophic Interactions
Source: PLoS Biol. 2007 Sep 4;5(9):e235. doi: 10.1371/journal.pbio.0050235 (PMC1964773; doi:10.1371/journal.pbio.0050235)
Supplement: Supplementary file 2 [file pbio.0050235.sd002.doc]

**Protocol S2: Analysis of PRESS Experiments**

We show here that PRESS experiments involving modifications of predator steady-state abundance may give highly misleading results when confounded by the effects of prey evolution represented in model (1). The scenario we consider is a system at steady state rather than cycling. Then, to estimate how strongly the predator affects the prey, a PRESS experiment is conducted that uses additions or removals to modify the predator density.

In our general model (1), suppose first that there is no direct competition between predator and prey (i.e., the function *f* is independent of predator density), and that predator abundance is held constant at some post-PRESS value *Y.* If the system then reaches a new steady state, so that $\frac{dx_{1}}{dt}=\frac{dx_{2}}{dt}=0$, the first line of (1) then implies that

$f(\bar{X},\theta_{i})-p_{i}Yg(\bar{Q})=0, i=1,2$.

This, in turn, implies that

$\frac{f(\bar{X},\theta_{1})}{f(\bar{X},\theta_{2})}=\frac{p_{2}}{p_{1}}$,

and the steady state total prey abundance $\bar{X}$ is the solution to this equation. Hence, $\bar{X}$is independent of *Y*, so that the final effect of the PRESS on the steady-state combined abundance of both prey types is exactly zero. If there is direct competition between predator and prey, then we have instead

$\frac{f(\bar{X},Y,\theta_{1})}{f(\bar{X},Y,\theta_{2})}=\frac{p_{2}}{p_{1}}$.

The PRESS effect on steady-state total prey density thus reflects only the direct competition between prey and predator, not the effects of predation.

In the situation giving rise to cryptic cycles (i.e., $p_{1}\ll p_{2},\theta_{1}\approx\theta_{2}$), the Implicit Function Theorem guarantees that there will be a new steady state (i.e., values of $\bar{X}$ and $\bar{Q}$ satisfying ) at least for small perturbations in predator density, because the Jacobian matrix of the conditions as a function of $(\bar{X},\bar{Q})$ is nonsingular. However, there is no guarantee that the steady state is stable, and in fact it will typically be unstable. With predator density held constant, the prey dynamics are

$$\frac{dx_{i}}{dt}=x_{i}\left(f(X,Y,\theta_{i})-p_{i}Yg(Q)\right), i=1,2$$

where $X=x_{1}+x_{2},Q=p_{1}x_{1}+p_{2}x_{2}$. The Jacobian for this model, which determines the local stability near a steady state, has determinant $-Yg'(\bar{Q})(1-p_{1})(f_{X,1}-p_{1}f_{X,2})$

when the model is scaled so that $p_{2}=1$, where $f_{X,i}=\frac{\partial f(X,y,\theta_{i})}{\partial X}$ evaluated at the steady state. When $\theta_{1}\approx\theta_{2}$ the determinant is approximately $-Yg'(\bar{Q})f_{X,2}(1-p_{1})^{2}$, which is negative because $g'$ and $f_{X}$ are both negative, hence the steady state is a saddle point and locally unstable.

Instability of the coexistence steady state means that a PRESS manipulation sets off an "evolutionary cascade" in which one prey type excludes the other, potentially resulting in large changes in prey density. Figure 7 shows an example, in which a small manipulation causes exclusion of one prey type or the other. A decrease benefits the vulnerable prey type, while an increase gives the advantage to the initially rare defended prey type, leading to a large change in total prey density. Note that the long-term effect only depends on the direction of the change in predator density, not on the amount of change: a PRESS manipulation of any size leads to exclusion of one type or the other.
